# Manipulation gesture effect in visual and auditory presentations: the link between tools in perceptual and motor tasks

**DOI:** 10.3389/fpsyg.2015.01031

**Published:** 2015-07-22

**Authors:** Amandine E. Rey, Kévin Roche, Rémy Versace, Hanna Chainay

**Affiliations:** Laboratoire d'Etude des Mécanismes Cognitifs, Université Lumière Lyon 2Lyon, France

**Keywords:** embodied cognition, gesture, visual and auditory presentation, perceptual task, motor task, situated cognition

## Abstract

There is much behavioral and neurophysiological evidence in support of the idea that seeing a tool activates motor components of action related to the perceived object (e.g., grasping, use manipulation). However, the question remains as to whether the processing of the motor components associated with the tool is automatic or depends on the situation, including the task and the modality of tool presentation. The present study investigated whether the activation of motor components involved in tool use in response to the simple perception of a tool is influenced by the link between prime and target tools, as well as by the modality of presentation, in perceptual or motor tasks. To explore this issue, we manipulated the similarity of gesture involved in the use of the prime and target (identical, similar, different) with two tool presentation modalities of the presentation tool (visual or auditory) in perceptual and motor tasks. Across the experiments, we also manipulated the relevance of the prime (i.e., associated or not with the current task). The participants saw a first tool (or heard the sound it makes), which was immediately followed by a second tool on which they had to perform a perceptual task (i.e., indicate whether the second tool was identical to or different from the first tool) or a motor task (i.e., manipulate the second tool as if it were the first tool). In both tasks, the similarity between the gestures employed for the first and the second tool was manipulated (Identical, Similar or Different gestures). The results showed that responses were faster when the manipulation gestures for the two tools were identical or similar, but only in the motor task. This effect was observed irrespective of the modality of presentation of the first tool, i.e., visual or auditory. We suggest that the influence of manipulation gesture on response time depends on the relevance of the first tool in motor tasks. We discuss these motor activation results in terms of the relevance and demands of the tasks.

## 1. Introduction

Grounded and embodied cognition theories claim that knowledge is assembled in order to prepare for action (Wilson, 2002) and is grounded in sensory-motor systems (Barsalou, 1999). The cognitive processes that underpin the use of knowledge are thought to be deeply rooted in physical action, with close links existing between perception, action and the environment (Glenberg, 1997; Clark, 2008). Consequently, it has been suggested that seeing an object does not involve only the processing of its different sensory properties but also the activation (or simulation) of motor components related to the object's typical action/use (Barsalou, 2008). The present paper focuses on the following question: Does the activation of motor components always result in an influence on the current task or does this influence depend on the relevance of motor activation for the current task? The study reported here focused more specifically on manipulation gestures that are typically associated with a tool (e.g., cutting for a knife, screwing for a screwdriver). We first explored whether the facilitation effect in perceptual and motor tasks depends on the relevance of the manipulation gesture activated by the prime. Second, because real-life experience is inherently multimodal and depends on our knowledge or our environment (Slotnick, 2004; Jääskeläinen et al., 2007; Versace et al., 2014), we compared the facilitation of manipulation gestures in response to visual (static tools in this study) and auditory (dynamic action-related) tool presentations in both perceptual and motor tasks.

Certain data reported by neurological and behavioral studies involving perceptual tasks has partially confirmed the idea that motor components can be automatically activated. It has been suggested that seeing objects typically activates actions that are associated with these objects irrespective of the task. Some neuroimaging studies have lent support to this argument by showing that neural motor areas are activated by a visual presentation of tools even when there is no intention of acting upon them (Chao and Martin, 2000; Vingerhoets, 2008). At the behavioral argument, Ellis and Tucker (2000) argued that visually presented objects activate motor components that are appropriate for grasping these objects. They showed that even if participants did not have to use the objects, the response times in categorization tasks were slower in congruent conditions (when the grip potentiated by an object was the same as that required by the ongoing task) than in incongruent conditions (see also Tucker and Ellis, 1998). Some studies have reported that actions with tools directly activate representations of their typical manipulation and have suggested that knowledge about manipulation gesture is involved in the selection of appropriate action plans (Creem-Regehr and Lee, 2005; Jax and Buxbaum, 2010; Ranganathan et al., 2011). According to these authors, specific memorized movements include action knowledge about manipulation and use that is automatically activated when a tool is seen (Buxbaum and Kalénine, 2010).

However, some studies have reported results that indicate non-automatic motor effects. For instance, the study conducted by McNair and Harris (2012) showed that seeing a tool automatically activates the grasp component rather than the manipulation component of motor activity in order to prepare for possible future use of the tool. They tested this assumption by comparing congruent vs. incongruent grasp and congruent vs. incongruent manipulation gestures between a prime and a target (both presented as pictures on a computer screen). The participants' task was to recall the name of the previously seen tool from a choice of many other tool names. The results showed that only grasp congruency enhanced participants' accuracy when identifying the previously seen tool. Furthermore, Pecher (2013) showed that a concurrent motor task did not interfere with the processing of the motor components of manipulable tools. This author asked participants to perform a perceptual task based on the perceptual or motor components of the stimulus, while also performing a concurrent motor task (i.e., various movements with their free hands). For instance, the participants performed visual tasks on manipulable and non-manipulable objects (e.g., they had to indicate whether a photograph of a tool was the same as or a mirror image of a preceding photograph) while performing a concurrent motor task. The author assumed that if the processing of manipulable tools is based partially on the activation of motor components, a concurrent motor task should interfere with processing. However, this study, like certain others that have been conducted, revealed no difference between the perceptual processing of manipulable and non-manipulable tools in a concurrent motor task paradigm (see also Pecher, 2013; Quak et al., 2014).

One possible explanation of motor activation effects (automatic or not) could lie in the intention to act (Massen and Prinz, 2009). Indeed, it has been suggested that relevant motor components are selected depending on the intention of the actor (Allport, 1987), which may be absent in perceptual tasks. Intention to act determines the nature of the information that is relevant for processing and this information can be processed irrespective of the target of the action (Craighero et al., 1998; Bekkering and Neggers, 2002; Lee et al., 2013; Roche and Chainay, 2014). One possibility is that activation of tool knowledge is not automatic but selectively modulated by the purpose of the action. If this is indeed the case, tool manipulation knowledge will not be activated in full and only those aspects relevant for the present situation will be activated. Contrary to sensorimotor theories, ideomotor theories have proposed that, rather than being automatic, tool knowledge depends on the intention (Prinz, 1997; Hommel, 2009). The intention to act in a given situation activates certain motor components that result from similar tool uses in the past and that are associated with the current environmental (Massen and Prinz, 2009). Thus, the goal of the action could be taken into account at a very early stage during the planning of a movement, i.e., when the relevant information is selected for the planning and execution of the action (van Elk et al., 2010). For example, the study by Lindemann et al. (2006) focused on how tool manipulation knowledge is involved in the preparation for an action. Their results suggest that tool manipulation knowledge is not activated automatically, but is only activated when the subject intends to grasp the object in a typical way instead of just making a finger-lifting movement. In a more recent study by Ranganathan et al. (2011), participants had to interact in three different ways with a glass placed either upright or upside-down; by grasping it, touching it with a clenched fist, or grasping it with a magnetic implement. Shorter initiation times were found in the case of simple grasping and grasping with the magnetic implement when the glass was placed upright as opposed to upside-down. This effect was not present when the participants touched the glass with their fist. These results, together with those obtained by Lindemann et al. (2006), suggest that an object does not activate motor components automatically but instead does so in light of the purpose of the action and the possibilities and intentions of the person performing the action. Consequently, if there is no intention to manipulate the tool, manipulation gesture components remain irrelevant to the task. However, it is unclear whether a tool-associated manipulation gesture component can be activated when it is irrelevant to the task as in a physical identity judgment task.

The second aim of the present study was to examine activation of the manipulation gesture processes as a function of the presentation modality of the tool (i.e., visual or auditory). In the traditional approach in which motor activation is considered to include manipulation gestures, visual information seems: (1) to be the preferred basis for the efficient execution of actions (Jacob and Jeannerod, 2005; Milner and Goodale, 2008) and (2) to rely on processes different from those involved in recognition and action knowledge (Milner and Goodale, 1995; Buxbaum and Kalénine, 2010). Accordingly, the visual processing of a tool would be sufficient in order to select the appropriate manipulation gesture for executing the action (Jax and Buxbaum, 2010). On the other hand, in a grounded cognition perspective, motor components are thought to be activated by another modality such as auditory information. Indeed, in everyday life, some actions are accompanied by a specific sound. Grounded theories suggest that the typical use of a tool is part of our knowledge about it (Gallese, 2005; Barsalou, 2008). This type of activation is consistent with the suggestion made by Gallese (2000) that tools differ from other objects because knowledge about tools includes one particular usage (Creem-Regehr and Lee, 2005). According to sensorimotor theories, knowledge about tools comes from sensorimotor traces that result from previous experience with tools. According to this view: (1) the auditory modality, just like any other sensory modality, could form the basis for the activation of motor components (see also Trumpp et al., 2014) and (2) motor and perceptual processes, including recognition and action knowledge, share common processes (see also Helbig et al., 2006, 2010; Sim et al., 2014).

In the present study, we tested the possible effect of motor component activation as a function of manipulation gesture in perceptual and motor tasks. In both tasks, a first tool was presented to the participants just before the presentation of a second tool on which they had to perform a perceptual and a motor task. In all the experiments, we manipulated the factor of Gesture Similarity between the first and second tool in three conditions. The gesture used for the two tools could be Identical (same tool, same gesture), Similar (different tools but similar gesture) or Different (different tools and gestures). We assumed that the activation of a similar gesture for the two tools would facilitate the subject response (i.e., faster response times or initiation times) if manipulation gesture is activated by the presentation of a tool. In addition, different results should be observed depending on the demands of the motor task. Indeed, if motor components are activated when they are relevant to the task, participants should only respond faster in the Similar than in the Different condition in the motor task since the activation of motor components is not relevant for the perceptual tasks. In addition to the type of the task (perceptual or motor), we also manipulated the presentation modality (visual or auditory) of the first object across the experiments. By using familiar tools associated with a well-known sound during utilization, we assumed that the auditory presentation of tools could activate manipulation gesture components in the same way as a visual presentation. More specifically, the first and second tools were presented visually in a perceptual (Experiment 1) and a motor task (Experiments 1 and 2), whereas the first tool was presented auditorily in Experiment 3 in perceptual and motor tasks.

## 2. Experiment 1

### 2.1. Method

#### 2.1.1. Participants

Sixteen participants from the University of Lyon 2 took part in Experiment 1 (13 females, *M* = 20.06, *SD* = 2.05) after completing a written consent form. All of them reported themselves as right-handed and with normal or corrected-to-normal vision and audition. The study was approved by the local Ethics Committee.

#### 2.1.2. Stimuli

**Figure 1:**
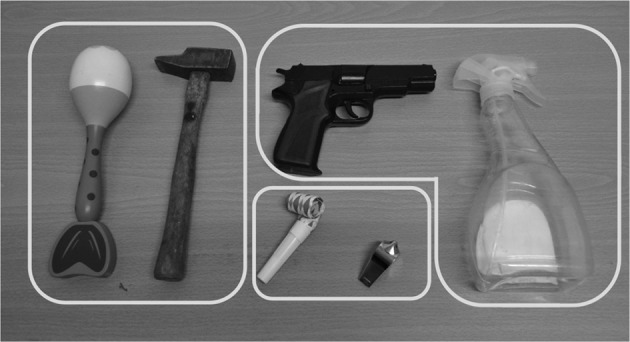
**The tools presented in the three experiments**. The groupings correspond to the pairs with simular utilization gestures.

The stimuli consisted of six manipulable objects which were presented as pictures in the perceptual tasks (in order remain consistent with the habitual perceptual paradigms, Labeye et al., 2008; Borghi et al., 2012; Pecher, 2013, for instance) and were physically in front of the participants for the motor tasks. As in the McNair and Harris (2012) study, they were subdivided into three pairs depending on the similarity of the gestures required for their use. The first and the second pairs corresponded to pistol/spraybottle and hammer/maracas (the maracas replaced the bell from the McNair and Harris study, because in the pre-test we found it to be too noisy when manipulated by the experimenter). The third pair—whistle/party blowout—was chosen on the same principle as the first and the second pair (see Figure 1).

The pictures were colored photographs of the six tools (2725 × 1187 pixels with a resolution of 300 × 300 dots per inch), taken from the same angle as that at which they were presented in the motor task. The photographs were presented at a distance of 65 cm from the participant's eyes.

#### 2.1.3. Tasks assignment and general design

The participants were tested individually in each of the tasks. The assignment of participants to the order of presentation of the perceptual and motor tasks was counterbalanced across participants. Before starting the experiment, we made sure that the participants knew the tools and the gesture associated with their use: the participants were first asked to say the name of the tools. If they failed to say the correct name, they were asked to describe the context of use (this was particularly useful for the party blowouts and spray cleaner since they have unfamiliar names in French). They were then asked to grasp the tool and demonstrate how to use it. If the gesture they made was only approximate, we told them normally we use it like this, showed them the correct movement and asked them to replicate the gesture (for example, maracas are moved front-to-back and not left-to-right). After this preparatory phase, the participants were asked to start performing the task to which they had been assigned.

In all the experiments presented in this study, we manipulated the factor of Gesture Similarity between the first and second tool over three conditions: (1) Identical: the first tool was identical to the second; (2) Similar: the first tool belonged to the same pair of tools as the second; (3) Different: the first tool was different and did not belong to the same pair of tools as the second (the first tool was chosen pseudo-randomly across the four remaining tools).

#### 2.1.4. Material and procedure

##### Perceptual task

###### Material

The experiment was conducted on a Macintosh IMac. OpenSesame software was used to set up and control the experiment (Mathôt et al., 2012).

###### Procedure

For the perceptual task, the participants were positioned facing the computer, with their right hand on the keyboard. Following the display of a fixation point, a first tool was presented to the participants for 1000 ms. After an Inter Stimulus Interval (ISI) of 500 ms, a second tool was presented. The second tool was displayed until the subject responded and was followed by an inter-trial interval of 1500 ms. The participants were asked to respond as quickly as possible by pressing the appropriate key on the keyboard (corresponding to the J and K keys, with the key assignment being counterbalanced across participants). After the phase of familiarization with the material, the perceptual task consisted of a physical identity judgment task (e.g., Vingerhoets et al., 2009). The participants had to indicate whether the tools were visually identical or different. Both tools were presented in one of the Gesture Similarity conditions (Identical, Similar, or Different). The first tool was always presented at 45° to the right (relative to the participant's midline), while the second tool was presented twice at 0° (aligned with the participant's midline) and twice at 90°, thus giving a total of 72 trials.

##### Motor task

###### Material

A Dell computer equipped with E-prime2 software (Psychology Software Tools, Inc., USA) was used to run the experiment and record initiation times. Liquid-crystal goggles (Plato Translucent Technologies, Toronto, ON, USA) were used to control the subjects vision and a home-made spherical trigger button of 4 cm diameter was connected to the computer and used to collect gesture initiation times. The tools were placed on a board measuring 40 cm by 50 cm.

###### Procedure

The participants were positioned facing the experimental board, with their right hand on the button. In order to be consistent with the other tasks, the primes and targets, i.e., the first and second tools, respectively, were presented on the experimental board one at a time at a distance of approximately 45 cm from the participant's hand and with their graspable component facing the participant. To avoid the affordance of exactly the same grasp movement between the first and second tools, we always used different orientations for the two tools. The orientations were 0° (aligned with the participant's midline) or 90° for the second tool, and always 45° to the right for the first tool.

**Figure 2:**
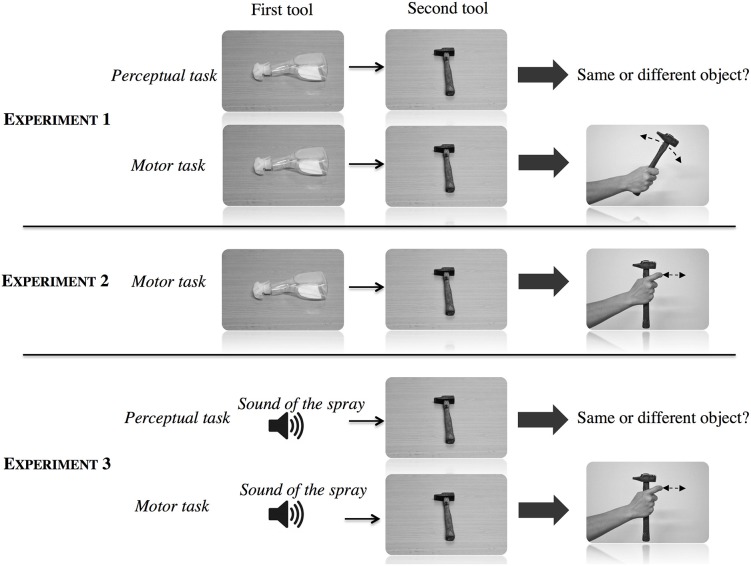
**Illustration of the perceptual and motor tasks in the three experiments**.

After 10 training trials in which all the conditions and tools were presented, each participant performed 72 trials which were identical to those used in the perceptual task (see Figure 2). The second tool was oriented at either 0° or 90° to create a variation in the grasp parameters and thus avoid repetitive grasp movements across trials. The trials were divided into 3 mini-blocks which were counterbalanced across participants.

All the trials started with a beep to remind the participant to place his/her hand on the release button. At the same time, the goggles became opaque for 1500 ms and a first tool was placed on the experimental board during this period. The goggles then became transparent for 500 ms so that the prime was visible, before turning opaque again for a further 1500 ms. During the ISI, the experimenter replaced the first tool on the experimental board with the second or, in the Identical condition, simply changed the orientation of the tool. At the end of the ISI, the goggles became transparent again and a simultaneous go signal indicated to the participant that he/she should grasp the second tool and show how to use it. The next trial then started with a beep. The participants were told to initiate the movement toward the tool as quickly as possible and simulate its use. They were given 3000 ms to do so.

To summarize, there were three differences between the tasks: the time interval between the stimuli (500 ms in the perceptual tasks and 1500 ms in the motor task), the presentation of the stimuli (pictures in the perceptual tasks and real tools in the motor task) and the nature of the task (comparison of two tools in the perceptual tasks and execution of the utilization gesture in the motor task).

#### 2.1.5. Statistical analyses

We measured Reaction Times (RT) and error rates in the perceptual tasks and Initiation Time (IT) in the motor task (IT corresponded to the time that elapsed between the go signal and the time when the participants removed their hand from the release button). Errors in the motor task were not analyzed due to their very small number (i.e., only three subjects made one or two errors in the motor task of the first experiment). Reaction (or Initiation) times that were greater than 1500 ms or less than 250 ms and also differed by more than 2.5 standard deviations from the individual participants mean for each condition were removed (less than 3% of the data). Preliminary analyses were conducted to check for normality (Shapiro-Wilks test) and sphericity (Mauchleys test) and no violations were found. We used the mean correct RT for the analyses. Separate analyses of variance (ANOVA) were performed for RT and error rates in the Perceptual task and for IT in the Motor task, with subjects as random variableand Gesture Similarity (identical, similar, different) as within-subjects factor.

Given that we tested specific hypotheses, planned comparisons were performed. A significance level of *a* = 0.05 was used for all the statistical analyses. Means and standard errors of RT/IT for all the tasks and experiments are presented in Table 1.

**Table 1:** **Means of reaction (initiation) times (in ms) for Experiments 1, 2, and 3 (between-subjects standard errors in parentheses)**.

**Experiments**	**1st tool modality**	**Task**	**Gesture similarity**		
			**Identical**	**Similar**	**Different**
Experiment 1	Visual	Perceptual	575 (38)	600 (41)	602 (40)
		Motor	521 (35)	528 (71)	520 (31)
Experiment 2	Visual	Motor	543 (23)	565 (27)	603 (29)
Experiment 3	Auditive	Perceptual	609 (27)	641 (31)	640 (29)
		Motor	458 (30)	476 (32)	490 (32)

For control purposes, we checked for a possible Tool Pair effect as well as for an interaction with the Gesture Similarity factor. We also checked for a possible Task Order effect and for an interaction between this and the Gesture Similarity factor. The data analyses were performed using STATISTICA (version 8.0, Stat-Soft, Inc.). The same analyses and controls were used for all the data presented in this article.

### 2.2. Results and discussion

#### 2.2.1. Perceptual task

The analysis of RT revealed a significant effect of Gesture Similarity, *F*_(2,30)_ = 4.49, *p* = 0.023, $\eta_{p}^{2}=0.23$. Planned comparisons showed that RT were faster in the Identical condition (*M* = 575 ms, *SE* = 38) than in either the Similar condition (*M* = 600 ms, *SE* = 41, *p* = 0.012) or the Different condition (*M* = 602 ms, *SE* = 40, *p* = 0.018), but no difference was observed between the Similar and Different conditions (*p* = 0.91). No simple effect of Task Order or Tool Pair was observed and there was no interaction between either Task Order or Tool Pair and Gesture Similarity (*p* > 0.1).

The analysis of error rates showed a significant effect of Gesture Similarity, *F*_(2,30)_ = 2.40, *p* = 0.029, $\eta_{p}^{2}=0.14$. The participants were more accurate in the Identical condition (*M* = 1.95, *SE* = 0.56) than in the Different condition (*M* = 3.22, *SE* = 0.59, *p* = 0.029. No difference was observed between the Similar (*M* = 2.77, *SE* = 0.54) and either the Identical, *p* = 0.20, or Different conditions, *p* = 0.42.

#### 2.2.2. Motor task

No simple effect of Task Order or Tool Pairs was observed, and neither of these interacted with Gesture Similarity (*p* > 0.1). No significant effect of Gesture Similarity was observed, *F*_(2,30)_ = 0.49, *p* = 0.62. We did not observe a significant difference between Identical (*M* = 521 ms, *SE* = 35 ms), Similar (*M* = 528 ms, *SE* = 71 ms) and Different (*M* = 520 ms, *SE* = 67 ms) conditions.

In the perceptual task, the fact that the two tools required a similar gesture did not facilitate the subjects response (no difference between the Similar and Different conditions was observed). The processing of the first tool seemed irrelevant for the processing of the second tool even if use of the two tools shared a similar manipulation gesture.

Surprisingly, no effect at all was found in the motor task. A previous study using an identical protocol, but with a grasping task, found priming effects when the prime and the target were identical tools (Roche and Chainay, 2013). One way to explain this difference compared to the present motor task is to consider that the movement is planned and controlled as a function of its purpose and that this determines the different steps involved in the movement, including the grasp (Rosenbaum and Halloran, 2006; Ansuini et al., 2008). If this is indeed the case, then it is possible that a priming effect will be found in a grasping task, whereas no such effect will occur in a task in which a tool-specific gesture guides the entire movement, (e.g., only grasping a toothbrush vs grasping a toothbrush and performing the tooth-brushing movement) (Massen and Prinz, 2009). Another possible explanation for this effect might be that the participants had learned that the first tool was irrelevant to the task and that they therefore ignored it. Indeed, Pfannmüller et al. (2012) have shown that visuomotor priming effects depend on the quality of prime processing and its memorization. It is possible that some of the processes involved in grasping are more likely to be activated automatically and are less intentional than those involved in the utilization gesture. As in the (Pfannmüller et al., 2012) study, we changed the protocol used for our motor task in Experiment 2 so that the first tool involved a preparation for a subsequent response. This ensured that the participants could not ignore the first tool as they could in Experiment 1. We asked the participants to grasp the second tool while performing the action corresponding to the first tool. This change of protocol increased the memorization and quality of prime processing (Pfannmüller et al., 2012). Thus, in this experiment, the intention to act was directed toward the second tool, whereas the planned gesture was determined in advance by the first tool.

## 3. Experiment 2

### 3.1. Method

#### 3.1.1. Participants

Sixteen self-reported right-handed participants took part in this experiment (8 women, *M* = 23.25, *SD* = 5.65). None of them had participated in Experiment 1.

#### 3.1.2. Stimuli and procedure

The same material and procedure as in the motor task in the previous experiment were used, except that we did not use a visuomotor protocol. In this experiment, the participants were told to grasp the second tool as quickly as possible while reproducing the action corresponding to the first tool, irrespective of the grasped tool.

#### 3.1.3. Statistical analyses

The same cutoff as in Experiment 1 was used (which eliminated less than 1% of the data). The IT werepre-processed according to the same criteria as in Experiment 1. ANOVAs were performed on the IT with subjects as a random variable and Gesture Similarity as within-subjects factor. In addition, the interactions of Tool Pair and Task order with Gesture Similarity were tested for control purposes.

### 3.2. Results and discussion

The results showed a significant effect of Gesture Similarity, *F*_(2,30)_ = 11.92, *p* < 0.001, $\eta_{p}^{2}=0.44$. IT were shorter for the Identical condition (*M* = 543 ms, *SE* = 23) than for the Similar (*M* = 565 ms, *SE* = 27, *p* = 0.026) and the Different conditions (*M* = 604 ms, *SE* = 29, *p* < 0.001). Moreover, IT were faster for the Similar condition than the Different condition (*p* = 0.011). The Tool Pair effect did not differ and did not interact significantly with Gesture Similarity (*p* > 0.1).

**Figure 3:**
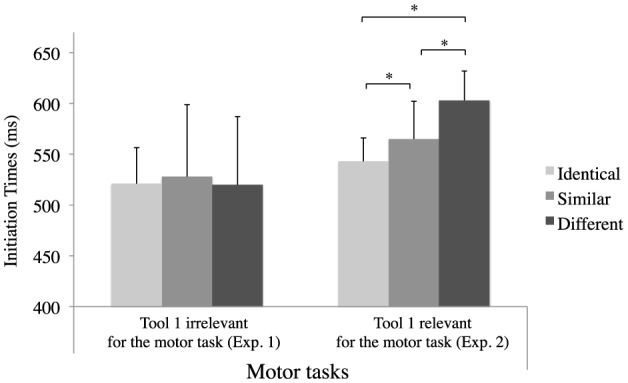
**Mean initiation times as a function of condition for the motor tasks in Experiments 1 and 2**. ^*^ *p* < 0.05.

To gain a better understanding of the relevance of the first tool for the task, which could explain the different patterns of results in the motor tasks in Experiments 1 and 2, we performed an ANOVA with the relevance of the first tool (relevant/Experiment 1 vs. irrelevant/Experiment 2) as group factor and Gesture Similarity as repeated-measure factor. The analysis revealed a significant effect of Gesture Similarity [*F*_(2,60)_ = 7.67, *p* = 0.002, $\eta_{p}^{2}=0.20$] and, more interestingly, showed that the interaction between Experiments and Gesture Similarity [*F*_(2,60)_ = 8.55, *p* < 0.001, $\eta_{p}^{2}=0.22$) was significant (see Figure 3). Planned comparisons are reported separately in the results section of each experiment.

In Experiment 2, we found an effect of Gesture Similarity. First of all, the results showed that movement IT were faster when the first and second tools were Identical rather than Similar or Different. We interpret this finding in terms of a facilitatory effect which enables subjects to plan their action in advance on the basis of the first tool presented just before manipulating the same (Identical) tool. Secondly, we found shorter IT in the Similar than in the Different condition. In both conditions, although the tools changed between the first and second presentation, their similarity in terms of motor manipulation nevertheless facilitated the initiation of the movement. In addition, and unlike in the motor task in Experiment 1, increasing the memorization and quality of the processing of the first tool enabled us to obtain an effect of Gesture Similarity. It seems possible that, unlike in a perceptual or grasping task, a more complex action such as demonstrating the actual utilization of a tool demands more situated processing. More generally, the results of Experiment 2 suggest that it is the intention to act that determines the processing of motor components (Allport, 1987) in the light of the overall goal of the action (Massen and Prinz, 2009).

To extend our study, Experiment 3 explored the possibility that a facilitation effect might be observed in response to an auditory presentation of the tool. If all the sensory-motor components are activated during the situation, then this activation should be induced by any sensory modality (e.g., the sound of a hammer should allow access to its action in just the same way as a hammer presented visually). Consequently, the first tool was not presented visually but auditorily by playing the sound associated with its utilization. The participants performed the perceptual identity task from Experiment 1 and the motor task from Experiment 2.

## 4. Experiment 3

### 4.1. Method

#### 4.1.1. Participants

Sixteen participants took part in this experiment (13 females, *M* = 21.38, *SD* = 3.12). None of them had taken part in the previous experiments.

#### 4.1.2. Stimuli

The respective sounds of the six objects replaced the presentation of the first tool in the motor task and the presentation of the photographs in the perceptual task.

#### 4.1.3. Procedure

The same general material and procedure as in the first experiment were used for this experiment. The only difference concerned the modality in which the first tool was presented. The visual presentation in Experiments 1 and 2 was replaced by the corresponding sound of tool utilization. We kept the same exposure duration of 1000 ms for the first tool. The second tool was presented in the same way as in the previous experiments (pictures in the perceptual task and the physical tool in the motor task). In the motor task, the goggles did not become transparent during the presentation of the sound. As in Experiment 2, the participants were told to grasp the second tool as quickly as possible while reproducing the action corresponding to the first tool (which had been presented auditorily), irrespective of the grasped tool.

#### 4.1.4. Statistical analyses

The same cutoff as in the previous experiments was used (which eliminated less than 3% of the data in the perceptual task and 1% in the motor task). The RT/IT were pre-processed using the same criteria as in Experiment 1. The ANOVAs conducted on the RT and error rates, and IT were performed with subjects as random variable, and with Gesture Similarity as within-subjects factor. In addition, the interaction of Tool Pair and Task Order effects were tested with Gesture Similarity as control.

### 4.2. Results and discussion

#### 4.2.1. Perceptual task

A significant effect of Gesture Similarity was observed on RT, *F*_(2,30)_ = 3.81, *p* = 0.033, $\eta_{p}^{2}=0.19$. Planned comparisons showed that RT were faster for the Identical condition (*M* = 609 ms, *SE* = 27) than for either the Similar condition (*M* = 641 ms, *SE* = 31, *p* = 0.05) or the Different condition (*M* = 640 ms, *SE* = 29, *p* = 0.017). However, no difference was observed between the Similar and Different conditions (*p* = 0.94). No simple effect of Task Order or Tool Pair was observed and neither Task Order nor Tool Pair interacted with Gesture Similarity (*p* > 0.1).

The analyses of error rates revealed no significant difference between the identical (*M* = 1.91, *SE* = 0.57), similar (*M* = 1.78, *SE* = 0.53) and different conditions (*M* = 2.55, *SE* = 0.53), *p* = 0.58.

#### 4.2.2. Motor task

**Figure 4:**
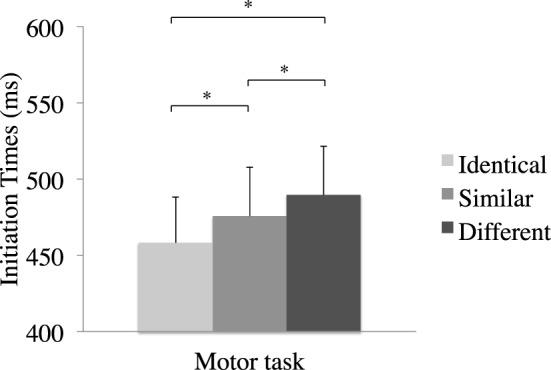
**Mean initiation times as a function of the condition for the motor task in Experiment 3**. ^*^ *p* < 0.05

The IT as a function of Gesture Similarity revealed a significant effect, *F*_(2,30)_ = 10.91, *p* < 0.001, $\eta_{p}^{2}=0.42$ (see Figure 4). Planned comparisons showed that IT were faster for the Identical condition (*M* = 458 ms, *SE* = 30) than for the Similar (*M* = 476 ms, *SE* = 32, *p* < 0.03) and the Different condition (*M* = 490 ms, *SE* = 32, *p* < 0.001), and that IT were faster for the Similar than for the Different condition (*p* = 0.05). No simple effect of Task Order or Tool Pair was observed, and neither Task Order nor Tool Pair individually interacted with Gesture Similarity (*p* > 0.1).

In the perceptual task, which in this case involved an auditory presentation of the first tool, the same pattern of results was observed as in the perceptual task of Experiment 1.

In the motor task, the results revealed shorter movement IT when the two tools were Identical than when they were Similar or Different. This result showed that there was a facilitatory effect on the planning of an action with the second tool when the participants had heard the same tool before. Moreover, and in line with our assumption, the effect of Gesture Similarity (previously observed in the motor task of Experiment 2, in which a similar protocol was used) was also observed when the two tools were similar. In fact, the participants responded faster in this condition than in a condition in which the tools were different. The difference between these two conditions lay in the similarity of the motor manipulation between the two tools in the Similar condition.

#### 4.2.3. Comparison of visual and auditory conditions in perceptual and motor tasks

We ran supplementary analyses to compare the visual and auditory modalities in the perceptual (visual modality in Experiment 1 vs. auditory modality in Experiment 3) and motor tasks (visual modality in Experiment 2 vs. auditory modality in Experiment 3). Separate ANOVAs were performed on RT/IT for the perceptual and motor tasks, with subjects as random variable, Gesture Similarity as within-subjects factor and Modality (of the first tool) as between-subjects factor.

Concerning the perceptual tasks, a significant main effect of Gesture Similarity [*F*_(2,60)_ = 7.88, *p* < 0.001, $\eta_{p}^{2}=0.20$; Identical: *M* = 592, *SE* = 23, Similar: *M* = 621, *SE* = 25, Different: *M* = 621, *SE* = 25] was found, but no main effect of Modality (*p* = 0.44) and no interaction between Gesture Similarity and Modality (*p* = 0.92). In Experiments 1 and 3, the participants were faster in the Identical condition than either the Similar or Different conditions.

As far as the motor tasks are concerned, we found a significant main effect of the Gesture Similarity [*F*_(2,60)_ = 21.07, *p* < 0.001, $\eta_{p}^{2}=0.41$; Identical: *M* = 501, *SE* = 20, Similar: *M* = 521, *SE* = 22, Different: *M* = 547, *SE* = 24] and a main effect of Modality [*F*_(1,30)_ = 5.77, *p* = 0.022, $\eta_{p}^{2}=0.16$; Visual: *M* = 571, *SE* = 26, Auditory: *M* = 474, *SE* = 31]. Participants were faster in the auditory condition (Experiment 3) than in the visual condition (Experiment 2). However, no interaction between Gesture Similarity and Modality was observed [*F*_(2,60)_ = 2.41, *p* = 0.098].

## 5. Discussion

The present study investigated motor facilitation by presenting familiar tools that either did or not require the same gesture when manipulated. The activation of motor components should be reflected by shorter reaction times or movement initiation times when the two presented tools share a similar gesture. More specifically, we explored whether the facilitation induced by manipulation gesture congruency depends on the relevance of the first tool for response preparation. We asked our participants to perform both perceptual and motor tasks, with the motor task requiring the physical execution of the movement. The originality of the present study lies in the manipulation of the relevance of the first tool for motor preparation within a motor priming paradigm. We also investigated whether motor preparation would be induced by auditory stimulation, i.e., whether an auditory presentation modality of tools can influence the initiation of a corresponding gesture in the same way as visual presentation.

As far as the presentation modality of the prime, which might play a role in motor activation, is concerned, we focus our discussion on the perceptual tasks in Experiment 1 (visual) and Experiment 3 (auditory) and the motor tasks in Experiment 2 (visual) and Experiment 3 (auditory), which were identical except for the modality of presentation of the first tool. With regard to the difference between the “similar” and “different” modalities of Gesture Similarity in these experiments, we observed an effect of Gesture Similarity in the motor task but not in the perceptual task, irrespective of visual or auditory presentation of the first tool. It has been argued that vision is the preferred sense for tool use (Jeannerod and Jacob, 2005; Milner and Goodale, 2008). However, audio-motor interaction has also been explored in the literature. For instance, D'Ausilio et al. (2010) showed that the congruency between motor preparation induced by an auditory stimulation and the future motor state had different consequences on motor performance. The present results are consistent with the theoretical framework of embodied and situated cognition. According to this framework, individuals encode all the sensory components of the situation when they interact with the environment, with there being no difference between a static (in this experiment, visual) and a more dynamic, action-related (auditory) presentation (Versace et al., 2009). Behavioral research has shown that congruent motor interactions between object pairs facilitate perceptual processes such as object recognition (e.g., Helbig et al., 2006; Kiefer and Martens, 2010). The results of the present study support the idea that motor components such as manipulation gesture can be reactivated not only by visual presentation but also by auditory presentation.

The comparison of motor tasks revealed a main effect of modality, with faster initiation times being observed in Experiment 3 (auditory presentation of the first tool) than in Experiment 2 (visual presentation). This effect must be interpreted carefully as we did not observe an interaction between Modality and Gesture Similarity. However, the faster initiation times in response to auditory presentation can be explained by the multimodality of the presentations (an auditory presentation of the first tool and a visual presentation of the second tool). Indeed, the literature reports that multimodal objects are processed faster and more accurately than unimodal objects (Giard and Peronnet, 1999). In an embodied perspective, the sound of a tool refers to its direct utilization and may accelerate the activation of components of the manipulation gesture. This difference might also be related to the experimental design of the study. Indeed, the goggles were opaque throughout the entire auditory presentation of the first tool, whereas they were successively opaque/transparent/opaque during the visual presentation of the first tool. Thus, participants might have focused more on the sound and the task with the auditory presentation. To determinate whether this is indeed the case, it would be possible to perform a further study with the same experimental design, i.e., in which the goggles are also opaque, then transparent, and then opaque again during the auditory presentation of the object.

Action planning can affect perceptual processing (see Theory of Event Coding, Hommel et al., 2001). Consequently, the presentation of a tool (or another stimulus which is associated with a particular action) automatically induces the production of the same action by the system. However, in the present study, the gestures associated with the tools in the perceptual tasks in Experiments 1 and 3 were irrelevant to the task and there was no intention to act. In these perceptual tasks, we did not find any difference between the similar and different gesture conditions. These results are consistent with the suggestion made by Vingerhoets et al. (2009) that motor knowledge about tools, and especially about their manipulation (corresponding gesture), is not activated by simply seeing a tool. These authors also found that grasp motor components could be automatically activated by seeing a tool, a finding which is consistent with the observation of shorter reaction times in the identical gesture condition than in the similar and different gesture conditions in our perceptual task, as well as with other studies (Ellis and Tucker, 2000; Sumner and Husain, 2008; McNair and Harris, 2012). In the present study, we can not exclude the possibility that the participants did not pay attention to the first tool in Experiment 1. Further investigations (in which the participants cannot ignore the first tool) should help to determine whether the results are due to the relevance of the first tool or to the attention paid to the tool.

All our motor tasks involved an intention to act. However, while in Experiment 1 the first tool presentation was irrelevant to the task, the motor tasks in Experiments 2 and 3 required the participants to plan their movements as a function of the initially presented tool and to perform the gesture with the second tool. Thus, in Experiments 2 and 3, the results of the motor tasks revealed furthermore shorter initiation times in the similar compared to the different gesture condition. The different patterns of results between the motor tasks used in Experiment 1 and Experiments 2 and 3 showed that an intention to act is not the only source of motor component activation. Indeed, it seems that the motor components need to be relevant to the task if they are to induce motor facilitation, especially when the task demands a more complex activity than simply grasping and carrying the tool. Unlike grasping, which is the non-reducible first step for actions with tools, it seems likely that tool use requires more specific processing of the situation and of the individuals needs. The fact that, in complex motor tasks such as tool use, individuals process only specific, relevant information can be seen as economical at the level of cognitive resources (Randerath et al., 2013). Embodied cognition theories claim that knowledge about tools comes from previous sensory-motor experiences with them (e.g., Gallese, 2005; Binkofski and Buxbaum, 2013). However, the question remains as to whether conceptual knowledge about tools might include manipulation knowledge (Garcea and Mahon, 2012; Osiurak, 2014). It seems that the best way to address this question would be to explore it in relation to the intention to act on the tool and the relevance of the motor components in the current situation.

### Conflict of interest statement

The authors declare that the research was conducted in the absence of any commercial or financial relationships that could be construed as a potential conflict of interest.
